# Melanin is a plenteous bioactive phenolic compound in date fruits (*Phoenix dactylifera* L.)

**DOI:** 10.1038/s41598-022-10546-9

**Published:** 2022-04-22

**Authors:** Muneeba Zubair Alam, Tholkappiyan Ramachandran, Asha Antony, Fathalla Hamed, Mutamed Ayyash, Afaf Kamal-Eldin

**Affiliations:** 1 Department of Food Science, College of Agriculture and Veterinary Medicine, United Arab Emirates University, P.O. Box: 15551, Al-Ain, United Arab Emirates; 2 Department of Physics, College of Science and National Water and Energy Center United Arab, Emirates University, P.O. Box: 15551, Al-Ain, United Arab Emirates; 3 Department of Veterinary Medicine, College of Agriculture and Veterinary Medicine, United Arab Emirates University, P.O. Box: 15551, Al-Ain, United Arab Emirates

**Keywords:** Analytical biochemistry, Scanning electron microscopy, Antimicrobials, Natural products, Infrared spectroscopy, X-ray diffraction

## Abstract

Date palm fruits (*Phoenix dactylifera* L.) were found to contain high levels of allomelanin (1.2–5.1%). The melanin is localized in the tanniferous cells between the inner and outer mesocarp tissues of the fruit. The melanin, extracted with 2 M sodium hydroxide, consisted of amorphous graphene**-**like granular structures of irregular shape and variable size. The date fruit melanin mainly comprises carbon (64.6%) and oxygen (30.6) but no nitrogen, and was thermally stable. It has radical scavenging (63.6–75.1 IC_50_, µg/mL), antimicrobial (250–1000 µg/mL), hypoglycemic (51.8–58.2%), and angiotensin-converting-enzyme inhibitory (65.8%) effects. The high level of melanin in date fruits highlights the importance of investigating its dietary intake and its impact on nutrition. This study also suggests that date fruit melanin can be a functional ingredient in foods, food packages, pharmaceuticals, and cosmetics.

## Introduction

Plant foods are highly appreciated as sources of health-beneficial dietary fibers and phenolic antioxidants^1^. Among plant foods, fruits are especially valued for their high contents and wide variety of phenolic compounds, including phenolic acids, flavones, flavonols, flavanones, flavanols, proanthocyanidins (also called condensed tannins), anthocyanins, and lignin. The different phenolic compounds exist in different forms such as monomeric aglycones, conjugated forms with various sugars and/or organic acids, complex polymers, and co-pigments. This broad diversity makes the simultaneous analysis of all phenolic compounds in plant foods with current analytical tools an almost impossible mission. This is mainly because no extraction solvent will quantitatively extract all forms that differ in polarity, size, and attachment to the matrix^2^. Polymeric phenolic compounds may be entirely ignored because of difficulties of extraction, which leads to underestimation of fruits total antioxidant potential and their contribution to nutrition and health.

Date palm is an important crop in warm to hot climates. A few studies that have addressed the analysis of extractable phenolic compounds from date fruit are summarized before^3^. Most of the extractions used in these investigations used simple solvents such as acetone, ethanol, ethyl acetate or methanol and assumed that the extractions were sufficient to recover the major phenolic antioxidants in the fruits^3^. However, these solvents cannot extract esterified or glycosylated phenolic compounds that are bound to the cell walls and other cellular components that require treatment with weak alkali and/or acids^3,4^. Our studies have observed that treating date fruits with weak alkali allows the extraction of hydrolysable phenolic compounds^3^. However, still, a significant amount of antioxidant activity remained in the pellet. We have also observed that a large antioxidant activity is released by extraction with strong alkali, which suggested the presence of phenolic compounds of melanin nature (unpublished results). A previous investigation reported the formation of melanin in dates fruits during storage by the polymerization of o-quinones through nonenzymatic browning reactions^5^.

Melanins are high molecular weight complex biomolecules, brown-to-black in color, found in various living organisms including humans, animals, plants, and microorganisms. Melanins are classified into animal eumelanin (black pigments) and pheomelanin (red and brown pigments) derived from the common precursor dopaquinone^6^ and plant allomelanin or phytomelanin (brown and black) formed from nitrogen-free precursors such as catechols and 1,8 hydroxynaphthalene’s^7^. Phytomelanin was described as dark insoluble pigments deposited between the outer and inner mesocarp in fruits of Angiosperms^8^. Natural melanin has a wide range of biological activities such as antioxidant, antimicrobial, anti-HIV, anticancer, anti-inflammatory, immunomodulatory, and anti-aging effects^9^. However, no studies have focused on dietary melanin and its contribution to human health.

This study reports the presence of high levels of melanin pigments in date fruits and provides evidence of their nature by conventional methods including UV–visible and Fourier transform infrared (FTIR) spectra, X-ray diffraction (XRD), X-ray photoelectron spectroscopy (XPS), and thermogravimetric analysis (TGA). We determined the variability in the level of melanin in 12 date cultivars and studied its antioxidant capacity (ABTS^·+^ and DPPH assays), antimicrobial effect against four bacterial strains (*Escherichia coli*, *Staphylococcus aureus*, *Salmonella typhimurium*, and *Listeria innocua*), hypoglycemic effects (inhibition of alpha-amylase and alpha-glucosidase), and antihypertensive activity (angiotensin-converting enzyme (ACE) inhibitory effect).

## Results and discussion

### Confirmation of the melanic nature of the pigment extracted from date fruits

The properties phenolic polymers extracted from date fruits were found to agree with the basic physiochemical identification tests for melanin^6^, specifically its insolubility in water and most organic solvents including acetic acid, acetone, ethyl acetate, ethanol, methanol, and chloroform, its solubility in high concentrations of sodium hydroxide solution (pH 12), sodium carbonate, and dimethyl sulfoxide (DMSO), and its resistance to degradation by acids and to bleaching by hydrogen peroxide, potassium permanganate, and other oxidizing agents^6,10^. The solubility of melanin in concentrated sodium hydroxide is explained by the conversion of hydroquinones to anionic forms while its solvation by DMSO is explained by the ability of DMSO to act as a hydrogen bond acceptor and its breakage of the inter-and intra-molecular hydrogen bonds within the melanin polymer^11^. When the date fruit melanin was precipitated from its alkali-soluble form by acidification, washed, and dried under a stream of nitrogen gas, it yielded a light brown powder. The melanin powders from the different date varieties had the following color values: lightness parameter (L*) ranging 16–38, redness (a* value) ranging 4–13, and yellowness (b* value) ranging 3–17.

**Figure 1:**
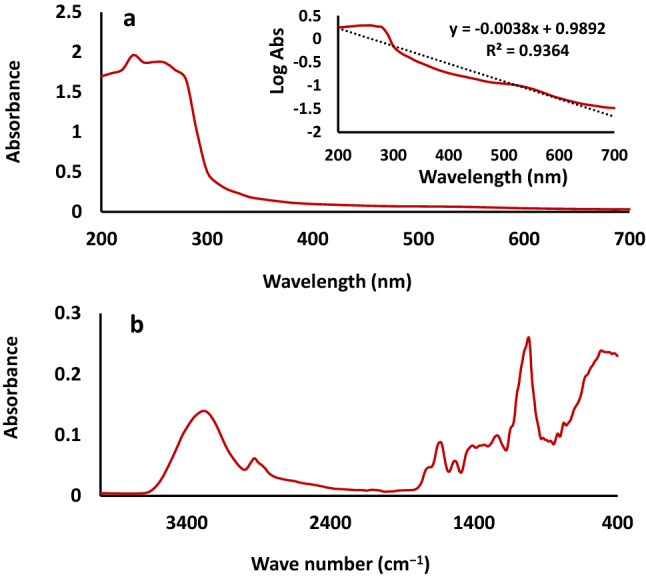
Absorption spectra of date fruit melanin (**a**) UV–visible and (**b**) Fourier-transform infrared (FTIR).

Figure 1a shows the UV–visible absorption spectrum of the date fruit melanin as a typical featureless broad-band monotonic spectrum, where light absorption is highest in the UV region (200–300 nm) and diminishes towards the visible region due to the complex structure indicating a high degree of conjugation in the molecule. This UV–visible absorption spectrum of melanin is different from the absorption spectra of organic molecules that are characterized by distinct absorptions of chromophores. This difference is explained by complex π-π* transitions with significant Mie and Rayleigh scattering and is related to their biological functions as photo-protectant and free radical sink^12,13^. The logarithm of the UV–visible absorption of the date fruit melanin has a negative slope (− 0.0038). Similar negative slopes of melanin, in the range of − 0.0015 to − 0.2646, were reported for melanin isolated from different sources^6^.

The FTIR spectrum of the date fruit melanin (Fig. 1b) is also consistent with previous literature on the identification of melanin^6^. The spectrum presents a characteristic -OH stretching vibrations band at 3600–3000 cm^−1^, C–H stretching vibration bands at approximately 2856 and 2926 cm^−1^, between 2950 and 2850 cm^−1^, and between 1465 and 1375 cm^−1^, specific stretches characteristic of phenyl groups and aromatic rings at 1500–1400 cm^−1^, phenolic –OH stretching vibrations at 1250–1180 cm^−1^, and aromatic C=C stretching vibrations at 1650–1600 cm^−1^. The fact that the shoulder at 1711 cm^−1^ is more apparent and distinct from the band at 1625 cm^−1^ provides evidence for the presence of C=C bonds while C–O deformation vibrations of aliphatic alcohols cause absorption bands between 1150 and 900 cm^−1^. The absorption bands below 900 cm^−1^ is related to wagging vibrations due to in-plane C-H bond deformation, aromatics C–H groups, and/or CH=CH substitution or conjugated systems^6,14^.

### Localization of the melanin pigments in date fruits

**Figure 2:**
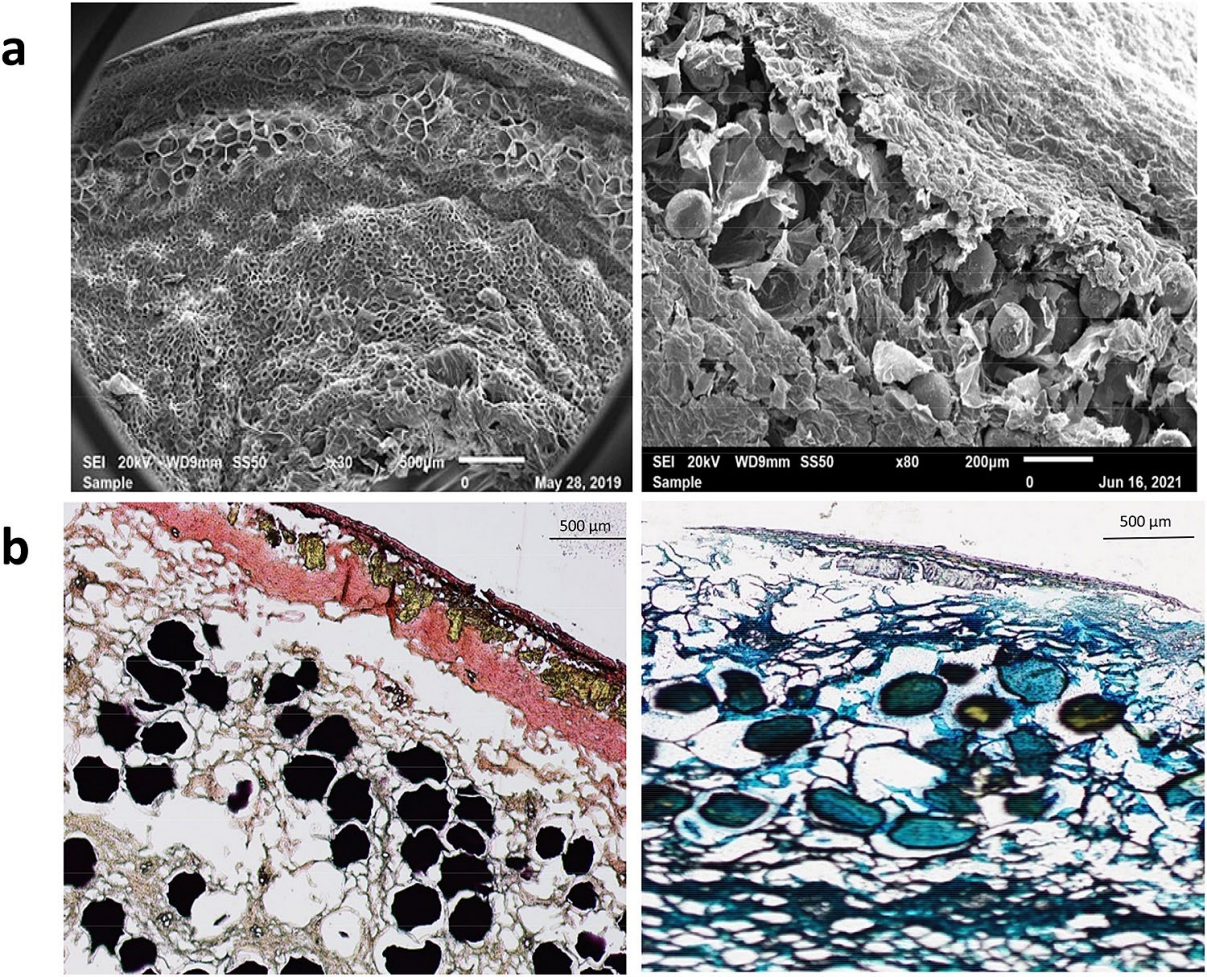
Localization of melanin in mature date fruits, (**a**) Scanning electron microscopic (SEM) image showing the tanniferous cells between the outer and inner mesocarp, and (**b**) Light microscopic images stained with 4-dimethyl amino cinnnamaldehyde (DMACA, left) and ferric chloride/potassium ferric cyanide (right) showing the presence the localization of melanin/proanthocyanidins in the tanniferous cells layer.

Figure 2a presents the SEM microscopic structure of mature date fruit (Tamar stage), showing that the mesocarp, which is composed of parenchymatous cells, is the largest tissue in date fruits and that it is separated into an outer mesocarp and an inner mesocarp by an intermediate layer of tanniferous cells^15^. Staining date fruit sections with dimethylamino cinnamaldehyde (DMACA) and ferric chloride/potassium ferricyanide confirmed that the major polyphenols in date fruit are concentrated in these relatively large tanniferous cells as shown in Fig. 2b. In their studies, Hammouda et al.^4,16^ suggested that the date fruits polyphenols are tannins (proanthocyanidins). However, DMACA staining is not selective for tannins, and it stains catechins and their derivatives with meta-hydroxy substitution in the A ring(s)^17^. The fact that the color of date fruits melanin was brown even in black varieties, such as Ajwa and Safawi, suggests that melanin and proanthocyanidins may co-exist in date fruits. The presence of condensed tannins of DP 10–17, based on (−)-epicatechin structure, has been reported in fruits of Tunisian Deglet Nour dates using high performance liquid chromatography^18^. It is well known that soluble tannins are responsible for the astringent taste of date fruits before full ripening and that their concentration decrease at the ripe stage, presumably due to oligomerization/polymerization^4^. Melanin and proanthocyanidins are biosynthetic products of catechins, and they may co-exist in the “taniferous layer” of date fruits. Further detailed studies are needed to identify the phenolic precursors and to study the relative prevalence and biosynthesis of melanin and proanthocyanidins (condensed tannins) in dates during fruit maturation.

### The levels of melanin in different date fruit varieties

**Figure 3:**
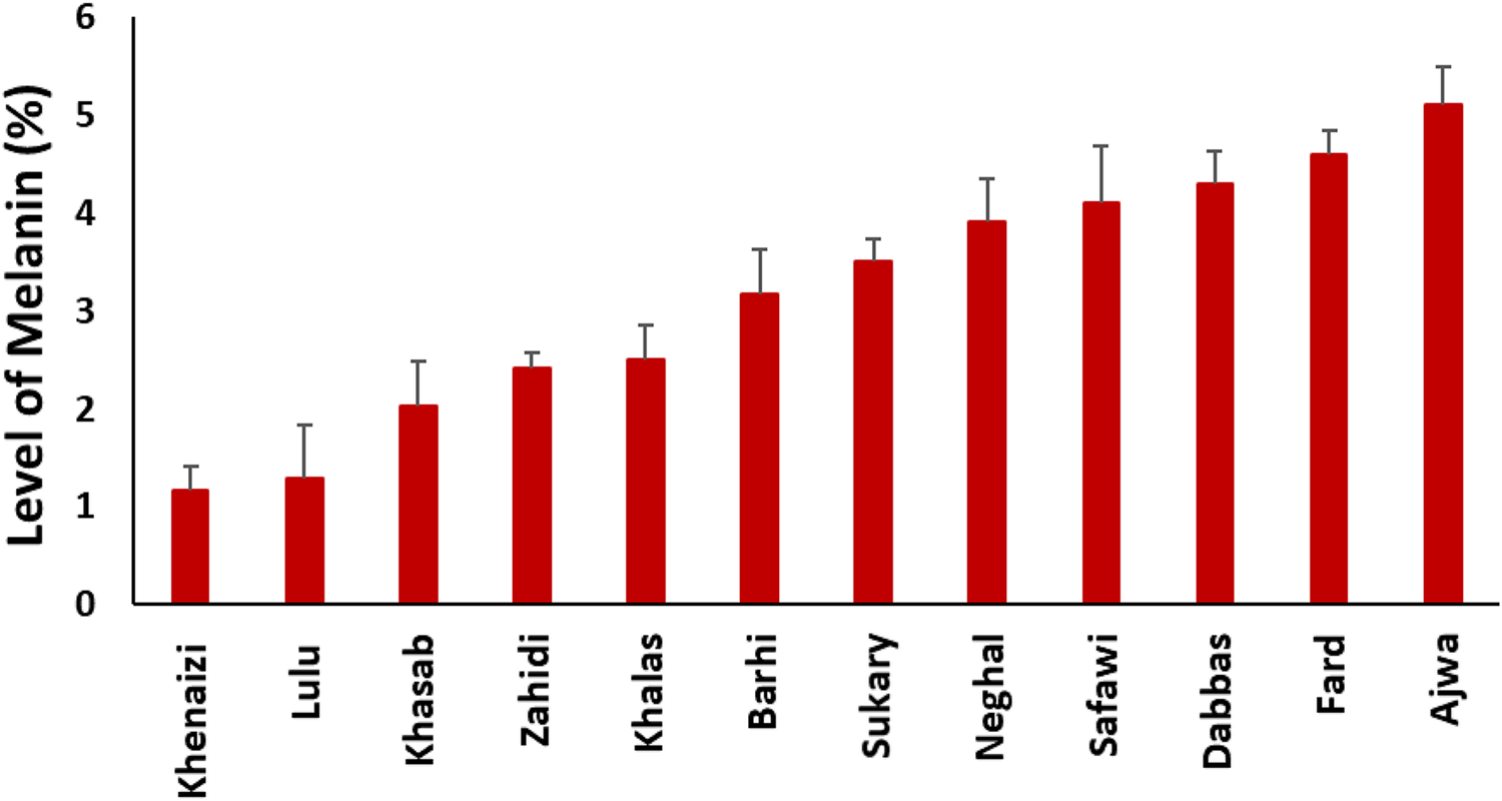
Levels of melanin in twelve date fruit varieties (bars represent standard deviation).

The levels of crude melanin in 12 different date fruit varieties extracted by ultrasound-assisted solubilization, drying, and gravimetric analysis ranged 1.2–5.1% on as is the basis (Fig. 3). The melanin extraction and quantification methods require optimization since several factors that affect the extraction of melanin, such as source of melanin,extraction method and its matrix as well as the localization intracellular/extracellular)^6^. Melanins present in natural sources are mostly found non-covalently bound to carbohydrates or proteins and require extraction methods that are able to break this bonding. Ultrasound-assisted extraction allowed an effective solubilization of melanin in alkaline solution compared to conventional methods^19^. This research suggests that the vast majority of phenolic compounds in date fruits is melanin and that its level is very high compared to levels of phenolic compounds believed to occur in foods. These high levels of antioxidants need to be considered when we evaluate their bioactivities and health potential. As we have suggested previously, “total” antioxidant activity methods may fail completely to describe the antioxidant potential of the wide range of phenolic compounds in fruits.

Only single samples of 12 date varieties were studied here to give an idea about the possible range of variability. Ajwa, Dabbas, Fard, and Safawi to have higher melanin contents but these results can not be considered conclusive and further research using a broader fruit collection (considering cultivars, cultivation location, and year) and an optimized quantification method are required for variety comparison. Several factors are expected to influence phenolic metabolism and melanin accumulation including genetic factors such as the genes controlling the color of the date fruit at the Bisr or Khalal stage, and environmental factors such as the exposure to sun light and heat and irrigation. In addition, the level of moisture in the fruit might interact with the solar radiations and heat and influence phenolic compounds metabolism. The effect of genetic and environmental factors on the soluble and insoluble phenolic compounds in date fruits needs to be investigated in the future.

### Physical characterization of date fruit melanin

**Figure 4:**
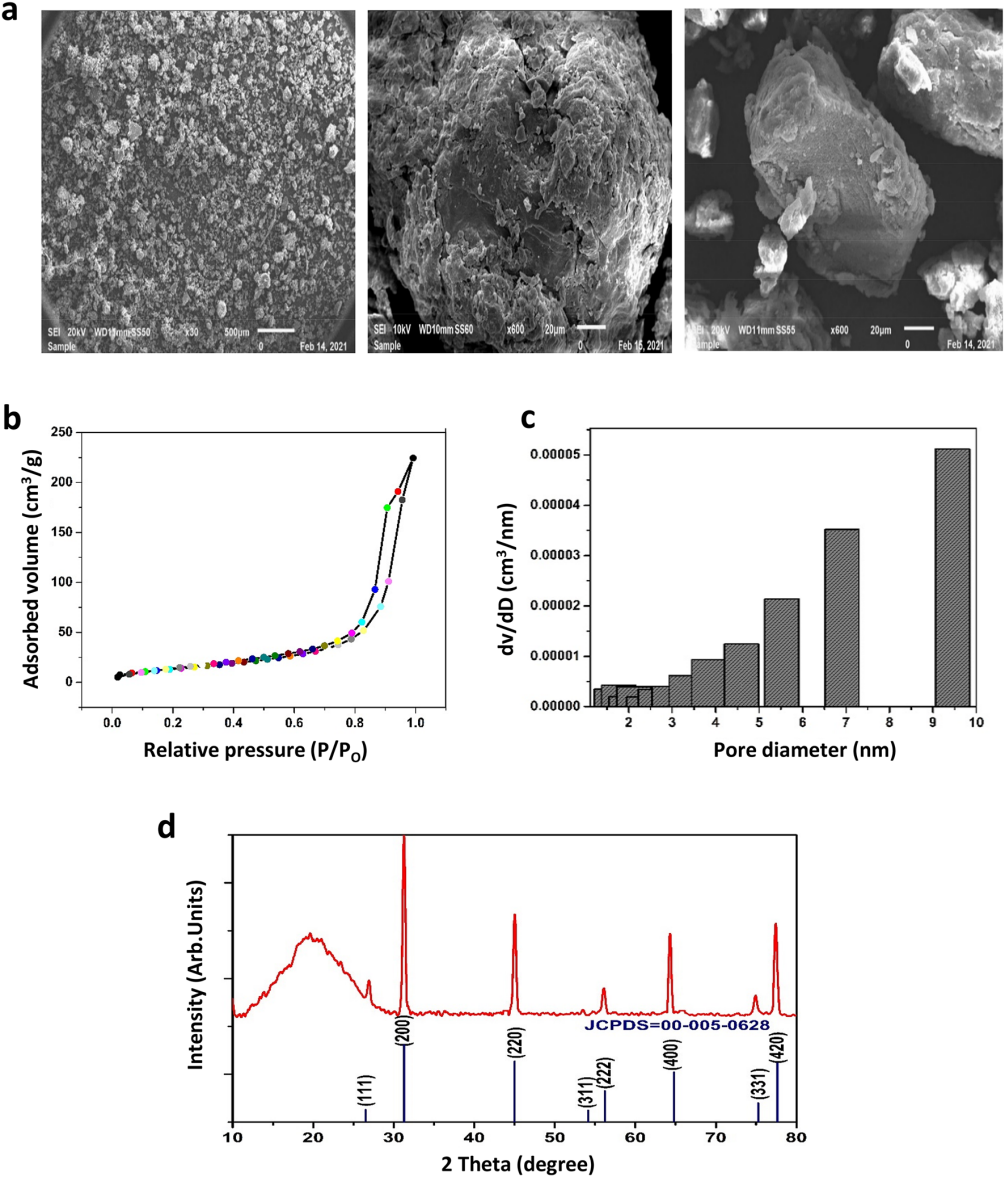
Date fruit melanin particle characteristics, (**a**) Scanning electron microscopic (SEM) Images of melanin particles at different magnification, (**b**) Sorption isotherm, (**c**) Relative Barret-Joyner Halender (BJH) pore size calculated from the sorption isotherm, (**d**) X-ray diffraction (XRD) profile showing the amorphous nature of the melanin. The crystalline components are artifacts from the sample preparation.

Scanning electron micrographs (SEM, Fig. 4a) show that the date fruit melanin particles consist of amorphous graphene**-**like granular structures of irregular shape and variable sizes (43–350 µm dimension), which agrees with nitrogen adsorption–desorption isotherms measurements of the specific surface area and pore size of the date fruit melanin. An adsorption isotherm provides information about the maximum amount of adsorbate that the material can have at a particular pressure. Figure 4b,c shows the BET isotherm and the relative Barret-Joyner Halender (BJH) pore size distributions obtained from the sorption–desorption isotherms, which can be described as type IV a with H3-type hysteresis loop according to IUPAC classification. The hysteresis loop's adsorption data in the relative pressure range (P/P_o_ = 0.47–0.98) indicates a mesoporous nature and the Brunauer–Emmett–Teller specific surface area was found to be 79.603 m^2^/g. The BJH pore size distributions showed that the sample had a narrow pore size distribution with pores between 0 and 6 nm. This is in agreement with the findings of Rahmani Eliato et al.^20^ who reported that the size of horsehair melanin pores is in the range of 3–5 nm. Such pore sizes and large specific surface area make date fruit melanin a suitable candidate for various applications such as a natural toxins absorber^21^.

The crystallinity and phase formation of the prepared melanin is characterized by X-ray powder diffraction (XRPD) analysis presented in Fig. 4d. The broad peak observed at 10°–30° supports the amorphous non-crystalline nature of the melanin sample^22^. The observed diffraction peaks correspond to crystal facets of (111), (200), (220), (311), (222), (400), (331), and (420) reflections, which represent the cubic structure of NaCl with a space group of Fm-3 m in good agreement with reference JCPDS card NO. 00-005-0628. No extra diffraction peaks were observed, indicating that the studied sample is amorphous and the presence of crystallinity is due to residual NaCl that is formed as an artifact during treatment with alkali and acid^23^.

**Figure 5:**
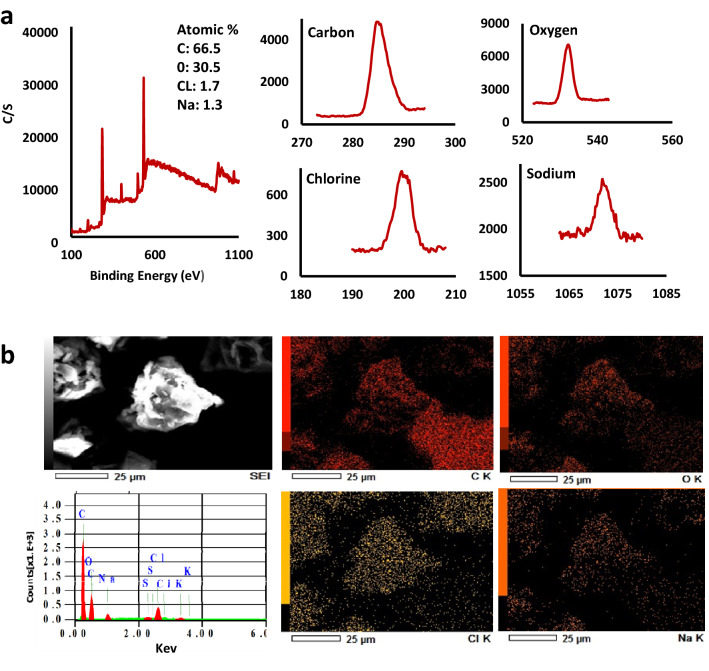
The elemental composition of date fruit melanin, (**a**) X-ray photoelectron spectroscopy (XPS), and (**b**) Energy dispersive spectroscopy (EDS), both showing carbon and oxygen as main components and small amounts of sodium and chlorine.

Figure 5 shows the results of X-ray photoelectron spectroscopy (XPS) and scanning electron microscopy/energy-dispersive X-ray spectroscopy (SEM/EDS) used to identify the elemental content in date fruit melanin. For XPS (Fig. 5a), the acquisition of the photoelectron intensity *versus* binding energy was performed from 1,200 to zero eV in vacuum to give no chance for skewed elemental composition^24^. The “O KLL” structure results from the excitation of Auger electron emission, which results from the relaxation of an ionized atom. The C–C, C–O, C=O, COOH groups have Binding energies in the range of 280–290 eV in the C region of XPS spectra. The Peaks in the O (1 s) region of the spectra occurred at energies in the range of 530 -535 eV, associated with COOH, C–OH, and C–O groups^25^. The XPS results showed that carbon and oxygen are the most abundant elements in date fruit melanin representing 64.6 and 30.6%, respectively.

The results of SEM/EDS analysis Fig. 5b agreed with the XPS analysis and showed that the other elemental components in the extracted date fruit melanin include NaCl artifacts of sample preparation and minor amounts of sulfur and potassium, which is in agreement with previous investigations^23^. The dominance of carbon and oxygen supports the presence of aromatic structures, the lack of nitrogen, and the very low level of sulfur support that date fruit melanin is an allomelanin.

**Figure 6:**
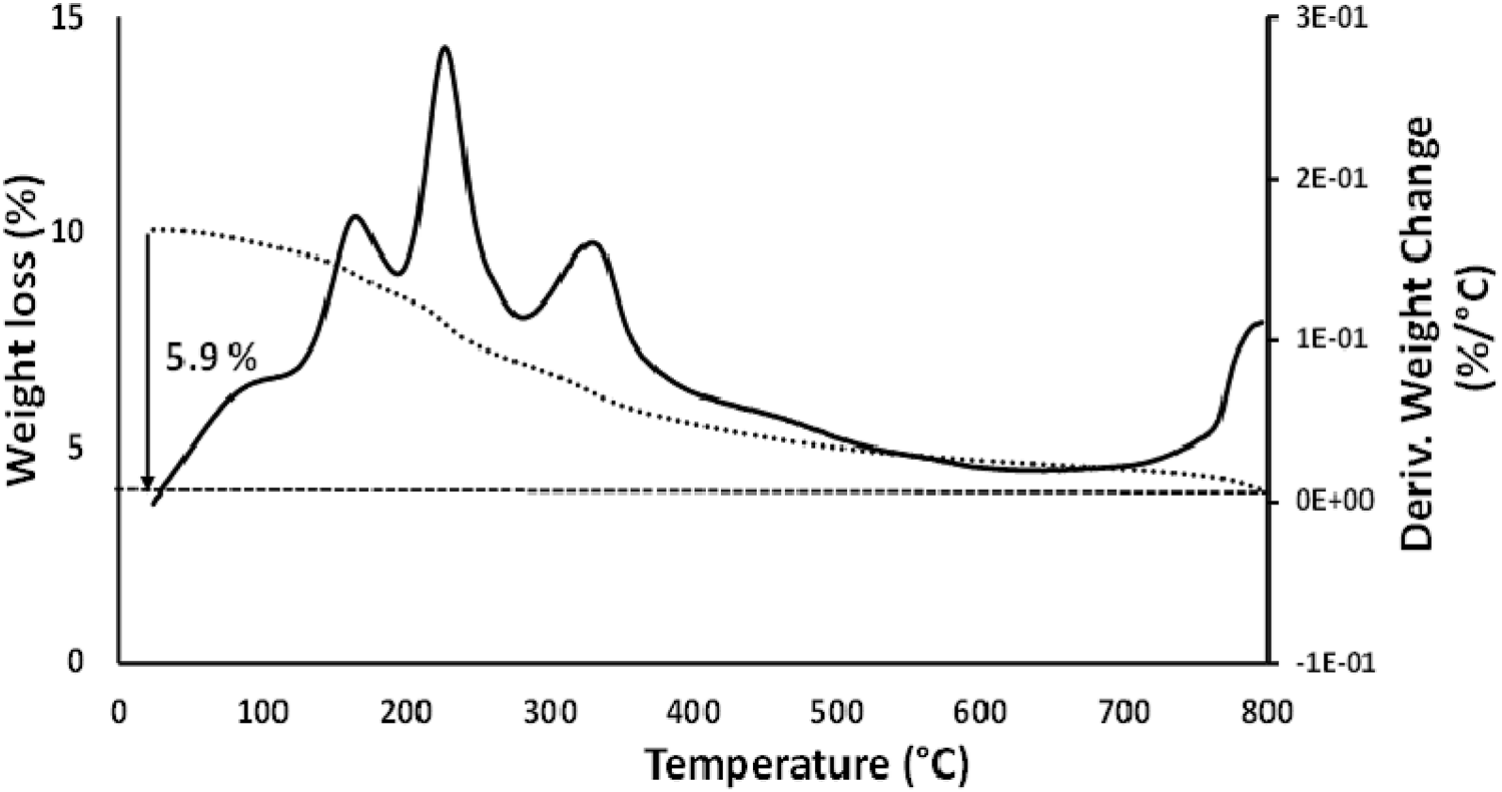
Date fruit melanin thermogravimetric analysis (TGA) represented by dashed line and differential thermogravimetry (DTG) by solid line.

Figure 6 presents the thermogravimetric analysis (TGA) results showing the thermal behavior of the date fruit melanin in terms of weight loss corresponding to the degradation temperature of the components in the sample. The melanin's derivative thermogravimetric (DTG) curves showed peaks representing the stages of thermal degradation of the main components in the samples. The exothermic peaks at 168 and 229 °C are due to the disintegration of melanin, and the broad peak around 332–335 °C with a shoulder is due to loss of carbon dioxide^26^. The thermal stability of melanin is evident because only 20% weight loss has occurred up to 220 °C, which is in agreement with Sajjan et al.^22^.

### High Performance Liquid Chromatography (HPLC)

**Figure 7:**
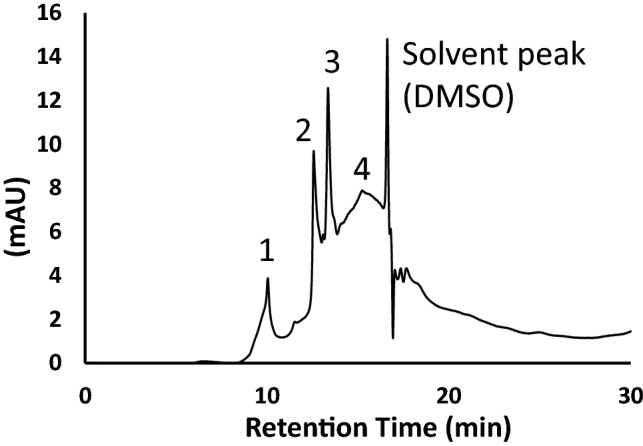
A typical HPLC separation of date fruit melanin on C18 reversed phase column using water: methanol; acetic acid (90: 10: 1, v/v/v) at o.2 mL/min as mobile phase and detection at 280 nm. Four peaks were detected with the last one eluting as a broad peak.

Figure 7 presents a typical separation of the date fruit melanin under the employed HPLC conditions. At least four peaks were detected with the last one eluting as a broad peak. The date fruit melanin peaks eluted close to the solvent peak, which agrees with previous findings showing that melanin is not retained on C18 reversed phase columns and elute as multi-peaks together with the solvent^27,28^. This result indicates a heterogenic character of the date fruit melanin in agreement with findings on melanin from other sources^29,30^. A heterogenic structure based on different precursors with different oxidation states and oligomerization patterns also supports the atypical, featureless broad UV–visible absorption spectra of melanin.

### Antioxidant and antimicrobial properties of date fruit melanin

The melanin samples showed more radical scavenging ability against ABTS^·+^ as compared to DPPH (Table 1), which may be due to different selectivity and diffusivity in these assays as suggested before^22^. Crippa et al.^31^ mentioned that melanin has various degrees of oxidation, which might affect its antioxidant potential. The chemistry of ABTS^·+^ and DPPH antioxidant assays follows single-electron transfer (SET, very fast), hydrogen atom transfer (HAT, slow), or their combination^3^. The scavenging rate increased with the increasing concentration of melanin in both assays.

**Table 1:** Antioxidant (%) by DPPH and ABTS, and antimicrobial activity (%) of date fruit melanin and sepia melanin (Data represented as Mean ± SD).

	Date fruit melanin	Sepia officinalis melanin
**Antioxidant activity (IC**_**50**_**, µg/mL)**		
ABTS	63.65 ± 0.23	45 ± 0.32
DPPH	75.12 ± 0.67	59 ± 0.88
**Antimicrobial activity (MIC and MBC, µg/mL)**		
*Escherichia coli*	250 ± 0.02	125 ± 0.05
*Listeria innocua*	1000 ± 0.06	1000 ± 0.01
*Staphylococcus aureus*	1000 ± 0.13	1000 ± 0.54
*Salmonella typhimurium*	1000 ± 0.07	1000 ± 0.55
**Hypoglycemic and ACE Inhibition activity (%,2000 µg/mL )**		
α-Amylase	58.2 ± 0.02	76.2 ± 0.01
α-Glucosidase	51.8 ± 0.05	78.4 ± 0.02
ACE	65.8 ± 0.02	88.3 ± 0.03

*IC_50_: half-maximal inhibitory concentration, MIC: Minimum inhibitory concentration, MBC: Minimum bacterial concentration, ABTS: 2,2′-Azino-bis (3-ethylbenzthiazoline-6-sulfonic acid sulfonic acid), DPPH: 2,2-diphenyl-1-picrylhydrazyl, ACE: Angiotensin-converting enzyme.

Gram-positive (*Staphylococcus aureus* and *Listeria monocytogenes*) and Gram-negative (*Salmonella typhimurium* 02-8423, and *Escherichia coli* 0157:H7 1934) foodborne pathogens were selected for the antimicrobial investigations. Except for *Escherichia coli* 0157:H7, all other bacteria have the same MIC and MBC value of 1000 µg/mL. The more lethal antimicrobial activity of melanin MIC and MBC value of 250 µg/mL against *Escherichia coli* indicates more disruptive action against the Gram-negative bacteria (Table 1). The MIC/MBC for all strains is 1, indicating that it has a potent bactericidal action. Antibacterial activities of melanin extracted from different sources such as horsehair^20^, watermelon seeds^32^ have also been reported. This indicates that date fruit melanin can be a potential bactericidal compound, but the mechanism of action requires further investigation.

### Hypoglycemic activity and ACE-inhibition

The date fruit and sepia melanin possessed good inhibitory effects on glycosidase and amylase activity as tested by α-amylase and α-glucosidase assays as shown in Table 1. The α-amylase and α-glucosidase activities of sepia melanin were slightly higher than those of date fruit melanin. Song et al.^33^ studied the hypoglycemic effects of sepia ink melanin obtained by alkali-assisted and enzymatic-assisted extraction. They found that melanin obtained by both extraction methods caused more than 50% inhibition at the concentration of 0.8 mg/mL. A number of phenolic compounds play important roles in modulating amylase and glucosidase activities such as epicatechins and proanthocyanidins^34^. Thus, melanin is a natural plant-based α-amylase and α-glucosidase inhibitor able to contribute to the management of postprandial hyperglycemia.

The results of ACE inhibition by date fruit and sepia melanin indicate that date fruit melanin is a potent ACE inhibitor (Table 1). In this study, date fruit melanin showed lower ACE inhibitory effect (66% at 2000 µg/mL) than monomeric phenolic compounds such as epicatechin (53% at 145 µg/mL) and apigenin (58% at 135 µg/mL)^35^.

## Conclusions

In this paper, we report the discovery of high concentrations of allomelanin (1.2–5.1%) in date fruits. This high level of a thermostable polymeric phenolic compound, with antioxidant, antimicrobial, hypoglycemic, and antihypertensive effects, is very interesting from a food technology as well as a health point of view. This fruit melanin is a promising source of edible antioxidant with a high potential for food and biotechnological applications. It may be used in different applications such as antioxidant supplements and additives in edible food packages and cosmetics. We are currently performing further research on the characterization of the chemical nature of the date fruit melanin and its exploitation possibilities.

This research highlights the need for further efforts in evaluating fruits and other plant foods as sources of polymeric antioxidants, especially melanin and proanthocyanidins that derive from catechin and epicatechin. The high level of melanin in date fruit supports our previous claim that total antioxidant activity methods are unable to account for the wide variety of phenolic antioxidants in foods partly due to insufficient extraction. Melanin and other high molecular weight phenolic polymers are not extractable in the solvents used for extracting phenolic compounds for the assessment of the “total” antioxidant activity, which raises an important question about the applicability of these methods.

Most of the research on the phenolic compounds in fruits have focused on those compounds that are extractable with organic solvents, mainly aqueous methanol^3^. To the best of our knowledge, studies have ignored the presence of unextractable phenolic polymers in fruits and that this study may open a new area of interest focusing on dietary melanin and its possible health implications as an antioxidant. Actually, there is some indication that different levels of non-polysaccharide constituents are included in the contents of dietary fiber in certain fruits since the content of dietary fiber obtained by gravimetric analysis is higher than that obtained by the analysis of polysaccharides and lignin^36,37^. The findings reported here encourage further investigations on the insoluble phenolic compounds in our diet and their connection with dietary fiber and its health benefits. It might recall the mechanisms behind the health-promoting effect of the “dietary fiber complex”. Even if melanin is not absorbed by the body, it might have important antioxidant and anti-inflammatory effects in the gastrointestinal tract.

## Materials and methods

### Date palm fruits

Fruits of ten Emirati date varieties (Barhi, Dabbas, Fard, Khalas, Khasab, Khenaizi, Lulu, Neghal, Sukary, and Zahidi) obtained from Al Foah Dates Factory (Al Saad, Abu Dhabi, UAE), and two varieties from Saudi Arabia (Ajwa and Safawi) purchased from the market (Al Ain, Abu Dhabi, UAE). Fruits were collected at the Tamar stage (full maturity) and stored at − 4 °C until analysis.

### Chemicals

Alpha-amylase from human salivary, alpha-glucosidase (1 unit/mL, Sigma), the ACE solution from rabbit lung, 2,2′-Azino-bis (3-ethylbenzthiazoline-6-sulfonic acid sulfonic acid) (ABTS^·+^), dibutylphthalate polystyrene xylene (DPX), 4-dimethylamino cinnamaldehyde (DMACA), 3,5-dinitrosalicylic acid (DNS), 2,2-diphenyl-1-picrylhydrazyl (DPPH), ferric chloride, hippuroyl-His-Leu hydrate, 6-hydroxy-2,5,7,8-tetramethylchroman-2-carboxylic acid (Trolox), *Melanin* from *Sepia* *officinalis*, p-nitrophenyl alpha-d-glucopyranoside (pNPG), phosphate buffer, potassium ferricyanide, potassium permanganate, potassium persulfate, potassium phosphate buffer, sodium borate buffer, sodium carbonate, sodium hydroxide, sodium potassium tartrate, starch, and xylene were from Sigma Chemical Company (St. Louis, Missouri, USA). Analytical-grade acetic acid, acetone, chloroform, ethanol, ethyl acetate, hydrochloric acid, hydrogen peroxide, and methanol were from Honeywell (Seelze, Hanover, Germany).

### Bacterial strains

The bacterial strains *Listeria monocytogenes* ATCC 7644, *Salmonella* Typhimurium 02–8423, *Escherichia coli* O157: H7 1934, and *Staphylococcus aureus* ATCC 15,923 were obtained from Prof. Richard Holley Laboratory, University of Manitoba, Canada.

### Ultrasound-assisted extraction of date fruit melanin

Date fruit samples (10 g) were soaked in 100 mL beakers containing NaOH solutions (75 mL, 2 M) and homogenized for 5 min using Ultra-Turrax T-25 (IKA, Staufen, Germany)^19^. Then, an ultrasound extraction was done using a Sonifier (SFX550, 1/2ʺ Dia Tapped disruptor horn, Branson Ultrasonics, Danbury, USA) at a frequency of 30 kHz and room temperature for 30 min. Thereafter, the mixture was centrifuged at 7240 Xg for 15 min at room temperature. After three consecutive extractions with intermittent centrifugations, the dark brown supernatants of alkali-soluble pigment were pooled and the pellet was discarded. The collected supernatant was treated with a concentrated hydrochloric acid solution to pH 2 and allowed to stand for 2 h followed by centrifugation (4070 Xg for 15 min at room temperature) to precipitate the melanin. The supernatant was discarded, and the crude pellet obtained was purified by organic solvents (chloroform, ethyl acetate, and ethanol) to remove lipids and other residues and repeated precipitations. The levels of dry melanin in each variety were determined gravimetrically, and the melanin was stored at 4 °C in the dark for further analysis.

### Confirmation of the melanin nature of the extracted pigment

The method of Hou et al.^38^ was followed for preliminary identification of extracted melanin. Solubility of date fruit melanin (10 mg) was tested in acetic acid, acetone, chloroform, distilled water, Dimethyl sulfoxide (DMSO), ethanol, ethyl acetate, methanol, 1 M hydrochloric acid, 1 M sodium hydroxide/sodium carbonate (20 mL each) after shaking and letting stand for 3 h. The pigment was also precipitated with 1% FeCl_3_^22^. The color of the melanin samples was measured as l*, a*, and b* color values using Hunter Lab colorimeter (Hunter Lab Inc., Reston, VA, USA). Melanin samples (0.5 g) were dissolved in NaOH (0.01 M 100 mL) to get 0.5% melanin solution, and after 5 min of sonication, its UV–visible spectra were scanned from the range of 200–700 nm using a BioTek EPOCH 2 Microplate spectrophotometer (Agilent, Santa Clara, USA)^39^. Attenuated total reflectance spectroscopy (ATR-FTIR) was performed using Spectrum Two IR coupled with Universal ATR (Perkin-Elmer inc., Norwalk, CT, USA). Date fruit melanin was placed on the ATR diamond crystal plate and scanned in the spectral range from 4000 cm^−1^ to 400 cm^−1^ at room temperature.

### Microscopic localization of melanin in date fruits

The localization of the melanin pigments in dates fruit was performed with light microscopy and scanning electron microscopy (SEM). For light microscopy, frozen date fruits were cut into pieces (*ca* 3 mm in length and breadth) prepared following^40^.The samples were dehydrated in a series of aqueous ethanol (40, 60, and 80%, for 30 min each) followed by immersion in 80% aqueous ethanol overnight to remove sugars. Next morning, the dehydrated specimens were rewashed in 80% ethanol, two washes in absolute ethanol (30 min each), and two washes in xylene (1 h each). Thereafter, the samples were embedded in paraffin followed by radial sectioning using Accu-Cut rotary microtome (SRM 200, Sakura Finetek, Japan). The sections were placed on glass slides, dried in an oven at 37 °C for 30 min, and the paraffin was removed in two changes of xylene (5 min each). Staining was performed by (i) the method of Hammouda et al.^16^ using drops of DMACA reagent (DMACA in ethanol (0.3% w/v) and 6 N HCl (1/1: v/v)) and incubation at 4 °C for 20 min, and (ii) the Schmorl method described by Shataer et al.^41^ where the slides were stained with an equi-mixture of 3% ferric chloride and 1% of potassium ferricyanide and incubated for 3 min at 4 °C. Rinsing of the stained sections was carried out several times with deionized water and the sections were dried and mounted with DPX. Light microscopy was performed on a MX40 Olympus Optical Microscope (Hamburg, Germany).

For date fruit SEM analysis, sample preparation to remove sugars and dehydration was performed following the method reported by George et al.^40^. The SEM images of mature date fruits and extracted melanin were obtained using Jeol Analytical Scanning Electron Microscope (JEOL JSM-6010PLUS/ LA, Tokyo, Japan) equipped with secondary electron imaging mode. The samples were mounted on aluminum stubs with silver paint as adhesive conductor and were sputter-coated with gold and observed at low vacuum using a power of 20 kV.

### Physical Characterization of date fruit melanin

The crystal structure of the melanin was investigated using a X-ray diffractometer (Shimadzu LabX XRD-6100, Kyoto, Japan). The X-ray diffraction (XRD) profile was recorded at room temperature and 30 kV with a current intensity of 30 mA over a 2θ range from 20° to 80° with a 0.02°/min step size using a Cu Kαradiation (1.541 Å) and results were compared to the International Centre for Diffraction Data (ICDD) PDF-2 database. X-ray photoelectron spectroscopy was conducted using Scanning XPS Microprobe (PHI Versa Probe III with monochromatized Al K_α1_ source). SEM/energy-dispersive X-ray spectroscopy (EDS) was also used for the elemental mapping of samples using the JEOL JSM-6010PLUS/LA Scanning Electron Microscope (Tokyo, Japan). The pore size, and specific surface area were studied using nitrogen sorption analysis in a QuadraSorb Station 1 Apparatus (Quantachrome, Boynton Beach, FL, USA). Isotherms were recorded at 77 K after degassing under vacuum for 10 h at 200 °C and room temperature. The surface area was determined using the Brunauer, Emmett and Teller (BET) method^20^.Thermogravimetric analysis (TGA) was performed on a Netsch STA409 EP (Selb, Germany) thermogravimetric analyzer. Melanin (10 mg) thermal stability was investigated by heating from 20 to 800 °C in a nitrogen atmosphere at a heating rate of 10 °C min^−1^. Differential thermogravimetric (DTG) curves and weight loss (%) were obtained from the TGA curve^26^.

### High Performance Liquid Chromatography (HPLC)

Dates melanin was suspended in dimethyl sulfoxide at 50 mg/ml and analyzed by HPLC as described by Sun et al.^27^ with some modifications. The chromatographic analysis was carried out on a Dionex rapid separation RS UPLC system equipped with DAD detector (Thermo Fisher Scientific, MA, USA) on an Ascentis C18 column (5 µm, 25 cm × 4.6 mm i.d., Supelco (Inc., Bellefonte, PA, USA) at 35 °C. The mobile phase was a mixture of water: methanol: acetic acid (90:10:1, v/v/v) and was run isocratically at 0.2 mL/min for 30 min. The injection volume was 20 µL and UV detection was carried out at 280 nm.

### Antioxidant activity of date fruit melanin

The radical scavenging activity of the date fruit melanin was determined using ABTS^·+^ and DPPH free radical methods. Melanin solution of various concentrations (20, 40, 60, 80, 100 μg/mL) was prepared in DMSO. ABTS^·+^ and DPPH radical scavenging activity was measured using the method previously published by Kang and Kim^43^. The percentage scavenging activity was calculated as follows

$$\%\;Scevenging\;activity = \left( {1 - Abs\;sample/Abs\;blank} \right)*100$$

where Abs sample is the absorbance of a sample at a given concentration, and Abs blank is the absorbance recorded for a blank.

### Antimicrobial effect of date fruit melanin

The antimicrobial activity of date fruit melanin was investigated by basically following the method of Al-Nabulsi et al.^44^ for minimum inhibitory concentration (MIC) and minimum bacterial concentration (MBC). Bacterial cultures (*Escherichia coli* 0157: H7 1934, *Staphylococcus aureus* ATCC 25,923, *Salmonella typhimurium* 02-8423, and *Listeria innocua* DSM 20,649) were prepared as per the method of Al-Nabulsi et al.^44^. Date fruit melanin and reference standard of sepia melanin were dissolved in DMSO at various concentrations (1.95–1000 µg/mL) and tested. The turbidity of the 96-well plate incubated at 37 °C for 24 h was observed at 600 nm using Epoch Spectrophotometer (BioTek EPOCH 2 Microplate, Agilent, Santa Clara, USA). To determine MBC, 100 μl suspension from the MIC wells was placed on BHI agar plate and incubated for 24 to confirm its bactericidal activity.

#### Hypoglycemic and antihypertensive activity

Alpha-amylase and alpha-glucosidase inhibition tests were done for date fruit melanin and sepia melanin as a reference standard prepared by dissolution in dimethyl sulfoxide (DMSO) to a concentration of 2000 µg/mL. The α-amylase assay was performed essentially following the method described by Yuan et al.^45^. Briefly, 200 μl of α-amylase from human salivary (1.0 unit/mL of phosphate buffer 20 mM, pH 6.9–7.0) was mixed with 200 μl of the melanin solution. After pre-incubation (37 °C for 5 min), the sample was mixed with 200 μl of starch (1%) in phosphate buffer (50 mL, 20 mM, pH 6.8). The mixture was left standing for 5 min before mixing with 200 μl of 1% DNS reagent (12% sodium potassium tartrate in 50 ml of 0.4 M sodium hydroxide). The mixture was heated for 15 min at 100 °C, diluted with 3 mL of distilled water in an ice bath, and the α-amylase activity was determined by measuring absorbance at 540 nm using Epoch Spectrophotometer (BioTek EPOCH 2 Microplate, Agilent, Santa Clara, USA).

For the alpha-glucosidase assay, the method mentioned by Yilmazer-Musa et al.^46^ was followed with slight modification. Melanin solution (50 μl) was mixed with α-glucosidase solution (100 μl, 1 unit/mL, Sigma, dissolved in 1 mL potassium phosphate buffer, 0.1 M, pH 6.8) and incubated at 37 °C for 10 min. Thereafter, *p*-nitrophenyl α-d-glucopyranoside (pNPG) (50 μl of 5 mM) was added as the substrate and the enzymatic reaction was performed for 35 min at 37 °C and stopped by adding 500 μl of 0.1 M Na_2_CO_3_. The α-glucosidase activity was determined by measuring the absorbance at 400 nm using Epoch Spectrophotometer (BioTek EPOCH 2 Microplate, Agilent, Santa Clara, USA).

The percentage inhibition for both the α-amylase and α-glucosidase assays was calculated as % Inhibition.

$$Inhibition \% = \left( {1 - Abs\;sample - Abs\;blank/Abs\;control} \right) \times 100$$

where Abs sample is the absorbance of a sample, Abs blank is the absorbance recorded for a solution without the substrate, and Abs control is the absorbance of a solution without the sample.

Liu et al.^47^ method was followed for the ACE inhibitory activity assay. Melanin solution, prepared by dissolution in DMSO to a concentration of 2 mg/mL (10 μl), was mixed with 10 μl of ACE solution (0.1 mU/mL) and pre-incubated at 37 °C for 30 min followed by incubation with 100 μl of the substrate (25 mM hippuryl-His-Leu in 50 mM sodium borate buffer containing 500 mM NaCl at pH 8.3) at 37 °C for 60 min. The reaction was stopped by adding 1 M HCl (85 μl) and hippuric acid was extracted with ethyl acetate (800 μl), dried for 30 min and the residue dissolved in distilled water(800 μl) and its UV spectra density was measured at 228 nm using Epoch Spectrophotometer (BioTek EPOCH 2 Microplate, Agilent, Santa Clara, USA).

$$ACE \; inhibition \% = Abs\;control - Abs\;sample/Abs\;control - Abs\;blank{ \times }100$$

where Abs control is the absorbance of the control, Abs sample is the absorbance of a sample, and Abs blank is the absorbance recorded for a blank.

#### Statistical analysis

Determining melanin contents in date fruits, antioxidant, antimicrobial, hypoglycemic, and antihypertensive activities were based on triplet determinations and expressed as mean ± standard deviation.

## Data Availability

All data generated or analyzed during this study are included in this published article.
